# Short-Term Exposure to PM_2.5_ and O_3_ Impairs Liver Function in HIV/AIDS Patients: Evidence from a Repeated Measurements Study

**DOI:** 10.3390/toxics11090729

**Published:** 2023-08-25

**Authors:** Hongfei Ma, Qian Zhang, Wei Liang, Aojing Han, Nianhua Xie, Hao Xiang, Xia Wang

**Affiliations:** 1 Wuhan Center for Disease Control and Prevention, 288# Machang Road, Wuhan 430024, China; 2 Qingshan District Center for Disease Control and Prevention, 4# Yangang Road, Wuhan 430070, China; 3 Department of Global Health, School of Public Health, Wuhan University, 115# Donghu Road, Wuhan 430071, China

**Keywords:** PM_2.5_, O_3_, constituents, hepatic enzymes, HIV/AIDS

## Abstract

Studies investigating the relationship between ambient air pollutants and liver function are scarce. Our objective was to examine the associations of acute exposure to PM_2.5_ and O_3_ with levels of hepatic enzymes in people living with HIV/AIDS (PWHA). Our study involved 163 PWHA, who were evaluated for serum hepatic enzymes up to four times within a year. We extracted daily average concentrations of PM_2.5_, PM_2.5_ components, and O_3_ for each participant, based on their residential address, using the Tracking of Air Pollution in China database. Linear mixed-effect models were utilized to assess the associations of acute exposure to PM_2.5_ and O_3_ with hepatic enzymes. Weighted quantile sum regression models were employed to identify the major constituents of PM_2.5_ that affect hepatic enzymes. The percent change of aspartate aminotransferase (AST) concentration was positively correlated with a 10 µg/m^3^ increase in PM_2.5_, ranging from 1.92 (95% CI: 3.13 to 4.38) to 6.09 (95% CI: 9.25 to 12.38), with the largest effect observed at lag06. Additionally, acute O_3_ exposure was related to increased levels of alanine aminotransferase (ALT), AST, and alkaline phosphatase (ALP) concentrations. Co-exposure to high levels of PM_2.5_ and O_3_ had an antagonistic effect on the elevation of AST. Further analysis revealed that SO_4_^2−^ and BC were major contributors to elevated AST concentration due to PM_2.5_ constituents. A stronger association was found between O_3_ exposure and ALT concentration in female PWHA. Our study found that short-term exposure to PM_2.5_ and O_3_ was associated with increased levels of hepatic enzymes, indicating that PM_2.5_ and O_3_ exposure may contribute to hepatocellular injury in PWHA. Our study also found that PWHA may be more vulnerable to air pollution than the general population. These findings highlight the relationship between air pollutants and liver function in PWHA, providing a scientific basis for the implementation of measures to protect susceptible populations from the adverse effects of air pollution. A reduction in the burning of fossil fuels and reduced exposure to air pollutants may be effective hazard reduction approaches.

## 1. Introduction

Ambient air pollution has become a worldwide public health issue that seriously affects human health [1]. Particulate matter and gaseous pollutants are the two primary constituents of ambient air pollutants [2,3]. According to *The Lancet*, in 2015, 4.2 million mortalities and 103,100,000 disability-adjusted life years were attributed to exposure to PM_2.5_ [4]. Air pollution is still responsible for roughly 9 million deaths each year, which is roughly equivalent to 1 in every 6 mortalities around the world [5]. Previous studies have shown that exposure to air pollution is associated with an increased risk of several diseases, such as cardiovascular disease [6], respiratory diseases [7], and metabolic disease [8]. Moreover, cumulative epidemiological evidence had confirmed that elevated pollution was linked to an increased risk of liver cancer [9], liver cirrhosis [10], and fatty liver disease [11]. However, to date, the effects of air pollutants on liver function have not received sufficient attention.

The liver performs an essential role in metabolizing exogenous substances, and its enzymes, including alanine aminotransferase (ALT), aspartate aminotransferase (AST), gamma-glutamyl transferase (γ-GGT), and alkaline phosphatase (ALP) [12], are often used to assess liver function [12]. Elevated hepatic enzymes can indicate hepatocyte damage and the potential presence of a liver disease [13]. A study of 150 newborns revealed that a 1 µg/m^3^ increment in PM_1_ and PM_2.5_ was associated with a 0.36 U/L (95% CI: 0.23, 0.49) and 0.29 U/L (95% CI: 0.18, 0.41) increase, respectively, in AST in the cord blood [14]. Another study in Korean adults showed that each 9 µg/m^3^ increase in PM10 increased logALT and logAST by 0.023 IU/L (95% CI: 0.016, 0.030) and 0.011 IU/L (95% CI: 0.006, 0.015), respectively [15]. Meanwhile, Qiu et al. also found that serum ALT concentrations were positively correlated with PM_2.5_ exposure in Chinese adults, while AST concentrations were negatively correlated with O_3_ exposure, but not with PM_2.5_ exposure [16]. These studies primarily focused on the long-term effects of air pollutants on hepatic enzymes in healthy populations, drawing inconsistent conclusions.

To date, no study has explored the impact of air pollution on hepatic enzymes in people living with HIV/AIDS (PWHA). As a vulnerable population, PWHA often suffer from a variety of opportunistic infections and frailty [17,18], and their hepatic enzymes levels may be lower than those of healthy individuals, owing to antiretroviral therapy (ART) [19,20]. Hepatic enzymes can be affected by a variety of factors, including medications and air pollutants [14,19]. Nonetheless, it remains unclear whether hepatic enzymes in PWHA are more susceptible to air pollutants that could further aggravate hepatic injury after the effects of medications have been eliminated.

Therefore, a panel study was conducted in Wuhan to assess the effects of short-term exposure to air pollutants (PM_2.5_, PM_2.5_ constituents, and O_3_) on levels of serum hepatic enzymes in PWHA undergoing the same treatment program. Through our findings, we aim to provide new insights into the effects of air pollution on liver function in this vulnerable population. Additionally, we hope to provide a scientific basis for the implementation of measures to protect susceptible populations from the adverse effects of air pollution.

## 2. Materials and Methods

### 2.1. Study Design and Sample

We recruited 163 PWHA who were 18 years of age or older to participate in a panel study [21]. All subjects were distributed across the 11 administrative districts of Wuhan and received treatment with highly active antiretroviral therapy (HAART), which consisted of a combination of a nucleoside reverse transcriptase inhibitor, a non-nucleoside reverse transcriptase inhibitor, and an intensified protease inhibitor. The Ethics Committee of the Wuhan Center for Disease Control and Prevention granted approval for this study (WHCDCIRB-K-2020001).

All subjects in the study completed an informed consent form before its commencement. From March 2020 to January 2021, hepatic enzymes of all the subjects were measured during each visit. After excluding patients with hepatitis, those using hepatotoxic medications (e.g., nevirapine, efavirenz, dolutegravir, raltegravir), and patients who had only one liver enzyme measurement, a total of 137 PWHA completed 528 follow-up visits. The process of the visit is detailed in Figure S1.

### 2.2. Hepatic Enzymes Measurements

At all follow-up visits, fasting venous blood samples were gathered from subjects before 10 AM. Venous blood samples (5 mL) were collected by medical staff from participants who had fasted for at least 8 h and packed into serum tubes, without an anticoagulant. The serum was obtained by centrifugation at 3000 RPM for 10 min within 30 min after blood collection. Serum ALT, AST, γ-GGT, and ALP concentrations were measured using an automated biochemical analyzer (HITACHI LABOSPECT 008 AS).

### 2.3. Exposure Assessment

Air pollutant data were obtained from the Tracking of Air Pollution in China database http://tapdata.org.cn/ (accessed on 20 November 2021) [22]. The daily PM_2.5_ concentrations and maximum 8 h average O_3_ concentrations were estimated, with complete spatial coverage, at 0.1° × 0.1° spatial resolution using a machine learning model and a gap-filling method, respectively. The methodology for predicting pollutant data had been previously published [22,23,24]. The predictive performance of the PM_2.5_ and O_3_ models was assessed using out-of-bag cross-validation and 5-fold cross-validation. The results showed that R^2^ values for daily PM_2.5_ and O_3_ prediction was 83% and 70%, respectively [22,23].

The chemical constituents of PM_2.5_, such as sulfate (SO_4_^2−^), nitrate (NO_3_^−^), ammonium (NH_4_^+^), black carbon (BC), and organic matter (OM), were obtained from operational CMAQ simulations, with PM_2.5_ components as constraints. To improve the accuracy of PM_2.5_ constituent measurements, a model was developed using observation data and the extreme gradient boosting algorithm to modify the relative contribution of PM_2.5_ constituents concentrations. The estimated PM_2.5_ constituents were in excellent accordance with the surface measurements, with R-values varying between 0.67 and 0.80 [24]. We geocoded each individual’s residential address for latitude and longitude using Google Maps. We then extracted air pollutants from the nearest grid cell in which each residential address was located and assigned them to the corresponding individual. In addition, the daily pollutant concentrations were computed based on the levels from the previous day up to the previous 7 days. Lag 0 represented the pollutant levels on the day immediately before. Lag01 was calculated from the mean pollutant levels of the previous 2 days.

### 2.4. Statistical Analysis

Linear mixed-effects models were employed to calculate the associations between air pollutants and levels of hepatic enzymes. The hepatic enzyme concentrations were subjected to a natural logarithmic transformation to approximate a normal distribution. Each model contains a random intercept to account for the results of repeated measures of association within each subject. The percent changes [exp (β) – 1 × 100] and 95% confidence intervals (CI) in the hepatic enzymes were calculated with a 10 µg/m^3^ increase in pollutant exposure. The covariates primarily included the general demographic characteristics and lifestyles of the participants, obtained from questionnaires completed at each follow-up visit, as well as ambient temperature data collected from the Wuhan Meteorological Center. The daily average temperature measured at the nearest monitoring site was matched with their respective home addresses. As described in prior research, it is necessary to adjust for variables that are both predictors of exposure and confounders of the exposure-outcome relationship [25]. In the final analysis, we adjusted for sex, age, body mass index (BMI), education (junior, senior, college, or above), occupation (office worker, manual worker, other), marital status (unmarried, divorced, married), annual income (<RMB 50,000, RMB 50,000–100,000, >RMB 100,000), disease history (none, hypertension, diabetes, cardiovascular disease, other diseases), smoking history (current, former, never), drinking status (current, former, never), and temperature as covariates. We treated the independent variables, and the covariates were applied as fixed effect terms. A natural spline curve, with a degree of freedom of three, was applied to fit the ambient temperature.

The synergy index (SI) was used to assess the interaction of co-exposure to PM_2.5_ and O_3_ regarding hepatic enzyme concentrations. We calculated the median exposure concentrations of PM_2.5_ and O_3_ for the 7-day average exposure at each follow-up. Individuals with exposure levels below the median concentration were defined as having low exposure, while those with exposure levels above the median concentration were regarded as having high exposure. All individuals were divided into four groups (PM_2.5_low-O_3_low, PM_2.5_low-O_3_high, PM_2.5_high-O_3_low, PM_2.5_high-O_3_high). SI was calculated as follows:

(1) $$\mathrm{SI}=\frac{\exp\left(\beta_{11}\right)-1}{\exp\left(\beta_{01}\right)-1+\exp\left(\beta_{10}\right)-1}$$

where β_01_, β_10_, and β_11_ were the effect estimates for the PM_2.5_low-O_3_high group, the PM_2.5_high-O_3_low group, and the PM_2.5_high-O_3_high group, respectively. SI < 1 indicates an antagonistic effect, and SI > 1 indicates a synergistic effect.

To examine the joint effect of PM_2.5_ constituents on hepatic enzymes, we transformed the concentration of the mixed constituents into quartiles and used weighted quantile sum (WQS) regression models to determine the degree of contribution from five constituents of PM_2.5_. The model was constructed using 40% of the available data, and the remaining 60% was validated using 1000 bootstrap samples to create the WQS coefficients. The WQS coefficients show the impact on the joint effect, while the weights indicate the contribution of each constituent to the overall effect [26].

We also selected the time window with the largest effect for stratified analysis to examine the potential covariate effects, using an interaction variable. Furthermore, sensitivity analyses were performed to confirm the reliability of our findings. First, to eliminate the impact of underlying diseases on the indicators, we excluded individuals with a history of disease. Second, we narrowed the sample down to those who were not currently consuming alcohol, in order to mitigate the impact of alcohol on the results. All statistical tests were conducted utilizing R software (version 4.1.4) and the significance threshold was set at 0.05, with two-tailed testing

## 3. Results

### 3.1. Description of Sample and Exposure

Table 1 provides an overview of the demographic characteristics of the 137 PWHA who participated in our study. The average age of the subjects was 47.64 ± 14.93 years, and the majority were males (93.43%). The participants’ average BMI was 22.13 ± 2.88 kg/m^2^. Of all participants, 42.33% were married, 53.28% had a personal income of less than RMB 50,000, and 48.18% had a college education or higher. Additionally, most of the PWHA were office workers (51.78%), non-smokers (60.58%), non-drinkers (66.42%), and had no history of illness (64.96%). The mean (standard deviation) levels of ALT, AST, ALP, and γ-GGT in PWHA at baseline were 30.52 (24.41) U/L, 69.54 (25.95) U/L, 96.08 (24.98) U/L, and 52.62 (39.91) U/L, respectively (Table 1).

The descriptions of the 7-day average levels of pollutants and the temperature are shown in Table 2. Throughout the follow-up period, the average (SD) levels of PM_2.5_, O_3_, and temperature were 39.8 (27.0) μg/m^3^, 100.7 (24.1) μg/m^3^, and 19.0 (8.5) °C, respectively. OM, NO_3_^−^, and SO_4_^2−^ comprised the majority of the total PM_2.5_ mass, and to a lesser extent, NH_4_^+^ and BC.

### 3.2. Air Pollutants and Hepatic Enzymes

**Figure 1:**
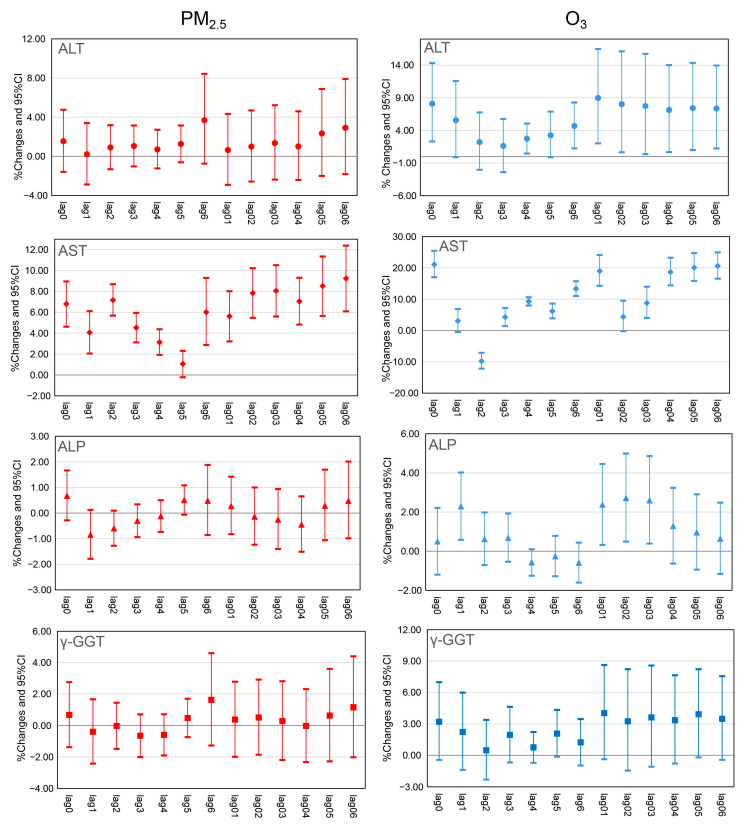
Associations between 10 µg/m^3^ increments of pollutants and hepatic enzymes in PWHA. All analyses were adjusted for sex, age, BMI, education, occupation, marital status, annual income, smoking history, alcohol consumption, disease history, and temperature. Abbreviations: PM_2.5_, particulate matter with an aerodynamic diameter of ≤2.5 µm; O_3_, ozone; ALT, alanine aminotransferase; AST, aspartate aminotransferase; ALP, alkaline phosphatase; γ-GGT, gamma-glutamyl transferase; CI, confidence interval.

The associations of PM_2.5_ exposure with ALT, AST, ALP, and γ-GGT concentrations are displayed in Figure 1. The percent change in AST concentration was positively correlated with a 10 µg/m^3^ increase in PM_2.5_, ranging from 1.92 (95% CI: 3.13 to 4.38) to 6.09 (95% CI: 9.25 to 12.38), with the largest effect appearing at lag06. However, no significant association of acute PM_2.5_ exposure was observed with ALT, ALP, and γ-GGT concentrations. We also found that residential O_3_ concentrations was positively related to the percent change in ALT (lag1, lag4, lag6, lag01–lag06), AST (lag0, lag3–lag6, lag01, lag03–lag06), and ALP (lag1, lag01–lag03) concentrations (Figure 1). The associations of O_3_ exposure with percent changes in ALT, AST, and ALP concentrations were the strongest at lag01, lag0, and lag02, with an increase of 8.96 (2.02, 16.47), 21.08 (17.02, 25.42) and 2.71 (0.49, 4.99) per 10 μg/m^3^ increase in O_3_ exposure, respectively. Meanwhile, no significant association was observed between acute O_3_ exposure and γ-GGT concentration.

Further analysis revealed that co-exposure to high levels of PM_2.5_ and O_3_ had an antagonistic effect on the increased percentage of AST concentration, with a synergy index of 0.568 (Table 3). It occurred at a 31.79%, 36.42%, and 19.58% increase in AST in the PM_2.5_high -O_3_high group, the PM_2.5_high-O_3_low group, and the PM_2.5_low-O_3_high group, compared to the PM_2.5_low-O_3_low group, respectively. However, the effect of the interaction of PM_2.5_ and O_3_ on levels of ALT, ALP, and γ-GGT was not observed in this study.

### 3.3. The Joint Effect Analysis of PM_2.5_ Constituents

**Figure 2:**
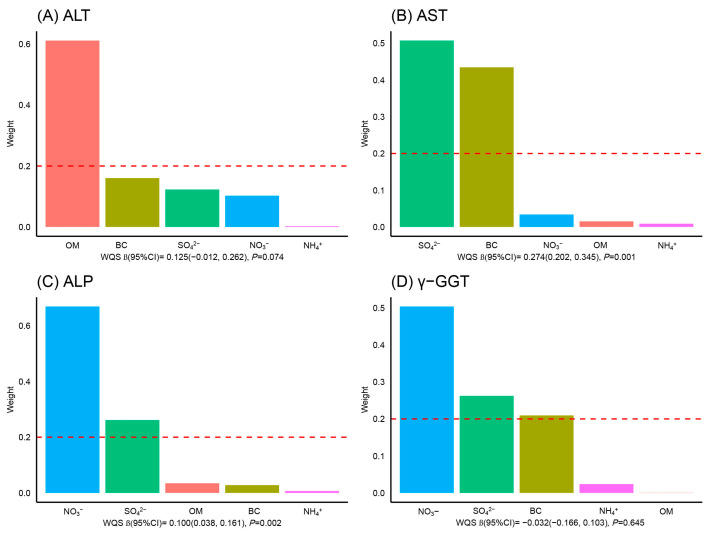
The weights of five constituents in PM_2.5_ with hepatic enzymes, based on WQS regression. All analyses were adjusted for sex, age, BMI, education, occupation, marital status, annual income, smoking history, alcohol consumption, disease history, and temperature. Abbreviations: PM_2.5_, particulate matter with an aerodynamic diameter ≤ 2.5 µm; SO_4_^2−^, sulfate; NO_3_^−^, nitrate; NH_4_^+^, ammonium; BC, black carbon; OM, organic matter; WQS, weighted quantile sum; ALT, alanine aminotransferase; AST, aspartate aminotransferase; ALP, alkaline phosphatase; γ-GGT, gamma-glutamyl transferase; CI, confidence interval.

The WQS regression models did not show any significant difference in the joint effect of PM_2.5_ constituents on serum ALT and γ-GGT concentrations (Figure 2A,D). However, we detected that mixed exposure to PM_2.5_ constituents was positively associated with the level of AST (β: 0.274, 95%CI: 0.202, 0.345), with the largest weight of contribution attributed to SO_4_^2−^ (0.51), followed by BC (0.43) (Figure 2B). Nevertheless, NO_3_^−^ was the predominant PM_2.5_ constituent responsible for the positive associations with ALP concentration (weight = 0.67) (Figure 2C).

### 3.4. Stratified Analyses

**Figure 3:**
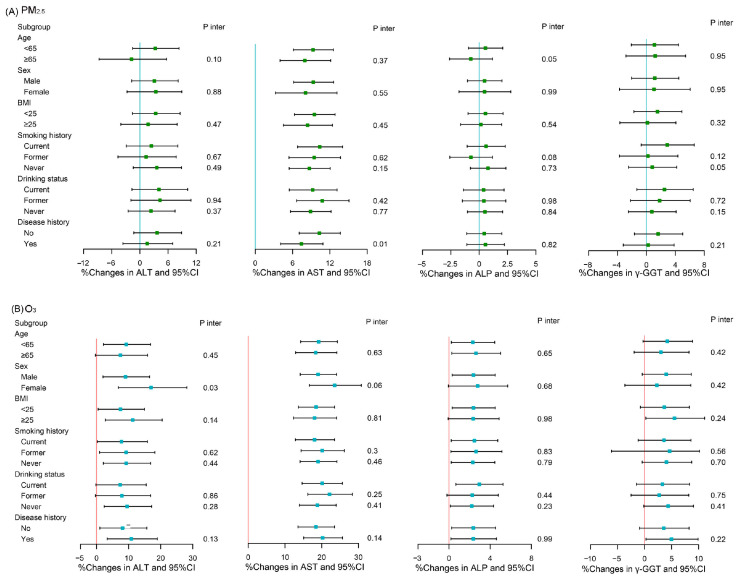
Interaction of covariates regarding associations of a 10 µg/m^3^ increment in air pollutants with hepatic enzymes in PWHA. Abbreviations: PM_2.5_, particulate matter with an aerodynamic diameter of ≤2.5 µm; O_3,_ ozone; ALT, alanine aminotransferase; AST, aspartate aminotransferase; ALP, alkaline phosphatase; γ-GGT, gamma-glutamyl transferase; BMI, body mass index; CI, confidence interval.

In the stratified analyses, we observed that PWHA without any disease history were more susceptible to PM_2.5_, which increased the level of AST compared to that of those with a disease history (*p* Interaction = 0.01) (Figure 3A). Moreover, the association of O_3_ with a percent change in ALT concentration was stronger in female PWHA compared with male (*p* interaction = 0.03) (Figure 3B).

### 3.5. Sensitivity Analyses

The sensitivity analyses showed that our finding were robust. The associations of PM_2.5_ and O_3_ with a percent change in ALT, AST, ALP, and γ-GGT concentrations were nearly unaffected, excluding PWHA with diseases history (Tables S1 and S2). Meanwhile, our results were still robust after excluding participants who were currently consuming alcohol (Tables S3 and S4).

## 4. Discussion

To our knowledge, this is the first study to explore the associations of PM_2.5_, PM_2.5_ constituents, and O_3_ with hepatic enzymes in PWHA. Our study determined that short-term exposure to PM_2.5_ was associated with a higher AST concentration. Likewise, acute O_3_ exposure was related to increased levels of ALT, AST, and ALP. PWHA may be more vulnerable to air pollution than the general population. Moreover, co-exposure to high levels of PM_2.5_ and O_3_ had an antagonistic effect on the increased AST concentration. We also revealed that SO_4_^2−^ and BC were major contributors to an elevated AST concentration. Female PWHA are more susceptible to acute PM_2.5_ and O_3_ exposures, resulting in elevated hepatic enzymes, due to their lifestyles, different hormone levels, and smaller airways.

Previous studies had reported associations between air pollutants and changes in hepatic enzymes among healthy populations. For instance, a panel study in the elderly demonstrated that each 13.2 µg/m^3^ increment in PM_2.5_ was associated with a 3% and 3.2% increment in AST and ALT concentrations, respectively [27]. Likewise, a longitudinal study including 318,911 elderly adults also reported that each 4.3 µg/m^3^ increase in PM_2.5_ was related to higher ALT and AST concentrations (β: 4.6%, 95% CI: 4.3%, 4.9%; β: 4.6%, 95% CI: 4.3%, 4.8%) [28]. Our results are in line with these findings. However, we discovered that the increase in serum AST concentration in PWHA after acute exposure to PM_2.5_ was greater than that in older populations (6.09% per 10 µg/m^3^ vs. 3% per 13.2 µg/m^3^). This finding suggests that PWHA may be more vulnerable to experiencing severe liver damage from acute exposure to PM_2.5_ due to their increased sensitivity. Similarly, a recent study demonstrated that short-term exposure to ambient PM_2.5_ could lead to reduced immunity in HIV/AIDS patients, which in turn leads to their increased susceptibility to air pollutants [21]. Additionally, Markevych et al. suggested that the mean concentration of γ-GGT increased by 5.1% for each 2.77 µg/m^3^ increase in PM_2.5_ among German populations [29]. Nevertheless, this association was not found in our study. The discrepancy in results may be explained by the different populations of the two studies and the larger sample size (5892 adults) in the Germany study.

Meanwhile, our study revealed that acute O_3_ exposure was related to an increase in ALT, AST, and ALP levels. Only a few studies explored the associations between O_3_ exposure and levels of hepatic enzymes. A study conducted in Chinese adults revealed a positive correlation between personal O_3_ exposure and ALT, and a negative correlation with AST [16]. Additionally, a panel study in the elderly observed positive associations of personal O_3_ exposure with ALT and AST concentrations [27]. Given the paucity of studies exploring the associations of O_3_ exposure with hepatic enzymes and the inconsistencies among the above studies, further studies are warranted to investigate associations between air pollutants and hepatic enzymes in PWHA.

We also found that PWHA in the PM_2.5_high-O_3_high group experienced a greater reduction in AST concentration than those in the PM_2.5_high-O_3_low group (36.42% vs. 31.79%). The reasons behind this phenomenon remain unclear. However, a study published in *Nature Geoscience* suggested that PM_2.5_ could inhibit O_3_ chemical production through non-homogeneous absorption of hydroperoxyl (HO2) radicals and nitrogen oxide (NOx) [30]. Moreover, it is well-known that PM_2.5_ and O_3_ can cause oxidative damage in humans, which may result in competition between the two pollutants for target organs [31,32]. Thus, it is plausible that the co-exposure to PM_2.5_ and O_3_ may have an antagonistic effect on hepatocellular injury.

The toxic effect of PM_2.5_ on human organs originates from its constituents [33]. Our study discovered that SO_4_^2−^ and BC in PM_2.5_ constituents were the two major contributors to elevated AST concentrations. Currently, studies on the associations between PM_2.5_ constituents and hepatic enzymes are lacking. However, an animal study showed that BC could cause inflammatory responses and apoptosis in mouse hepatocytes [34]. Moreover, long-term exposure to BC was related to an elevated incidence of liver cancer in six European cohorts [35]. Additionally, Takikawa et al. found that SO_4_^2-^ had a significant cytotoxic effect on hepatocytes [36]. BC is primarily sourced from transportation and industrial combustion, while SO_4_^2−^ is mainly derived from the photochemical conversion of sulfur dioxide emitted from fossil fuel combustion. As such, reducing the particulate matter generated by burning fossil fuels may be an effective approach for mitigating the adverse effects of PM_2.5_ exposure on liver function.

The mechanisms involved in the adverse effects of air pollutants on the livers of PWHA are not defined, but hypotheses suggest that inflammation could be a crucial factor [9,37,38]. Inhaled air pollutants are phagocytosed by immune cells (e.g., bronchial macrophages and hepatic macrophages), which release inflammatory cytokines into the blood, further leading to a hepatic inflammation response [39,40]. However, PWHA have an impaired immune system, which prevents them from activating an effective immune response when proinflammatory cytokines enter the bloodstream, leading to further increases in proinflammatory cytokines in the body [41]. The accumulation of a high level of proinflammatory cytokines will lead to damage or death of hepatic cells, eventually resulting in higher levels of hepatic enzymes being released into the bloodstream [42]. Another plausible explanation is the oxidative stress responsible for liver damage [43,44,45]. Both PM_2.5_ and O_3_ may influence the activity of peroxisome proliferator-activated receptors and alter lipid metabolism in hepatocytes and hepatic astrocytes [46,47]. Meanwhile, the long-term survival of HIV-1 in PWHA exacerbates cellular oxidative stress [48], which can produce large amounts of reactive oxygen species that may trigger hepatocyte damage and contribute to elevated levels of hepatic enzymes [49,50]. In general, the liver function of PWHA is more vulnerable to atmospheric pollutants. Toxicological experiments are required to further explore the mechanisms of air pollutants on hepatic enzymes.

Our study showed that the effect of O_3_ exposure on ALT concentration was more pronounced in female PWHA, which is in agreement with the results of the study conducted by Li et al. [28]. This susceptibility can be partly interpreted by the fact that women have a stronger inflammatory response when stimulated by pollutants, as well as the fact that women have different lifestyles and hormone levels, along with smaller airways [51,52,53,54]. This indicates that females should pay greater attention to the prevention of health hazards from O_3_ exposure. Moreover, PWHA with no disease history were more susceptible to PM_2.5_ exposure. The precise mechanism for the effect modification was not completely known. One possibility is that PWHA without underlying disease may spend more time outdoors engaging in physical activities, leading to increased exposure to PM_2.5_. Further studies with larger-scale sample sizes are required to verify our findings.

There are several limitations that need to be considered when interpreting our study results. First, spatiotemporal models were used to assess individual air pollutant exposure levels, with some misclassification bias compared to the use of individual samplers. However, spatiotemporal models of personal pollutant exposure are presently considered a dependable scientific approach. Second, since the TAP database only contains data for PM_2.5_, PM_2.5_ constituents, and O_3_, we only assessed the effects of these pollutant exposures on the hepatic enzymes of PWHA, without including other pollutants. Third, we were unable to restrict the therapeutic medications used by the PWHA to ensure uniformity, due to emergence of drug-resistance and changes in medications. Nevertheless, all subjects received ART therapy with the same treatment program to minimize bias caused by different medications. Fourth, we were unable to incorporate relative humidity data for adjustment in our model because there was a lack of humidity data available for the city of Wuhan. Fifth, this study was performed among PWHA, and we did not include healthy subjects as a control group. This limitation poses a constraint on our ability to draw a conclusive inference that PWHA are more prone to damage from air pollution in comparison to healthy subjects, during the same time frame. Therefore, in the future, we will include both healthy subjects and PWHA concurrently to investigate the effect of air pollution on liver function in both groups. Additionally, we will further elucidate the potential mechanisms of liver damage caused by atmospheric pollution by examining a broader range of biomarkers.

## 5. Conclusions

Our study revealed that short-term exposure to PM_2.5_ and O_3_ was linked to increased levels of hepatic enzymes in PWHA, indicating a greater likelihood of hepatocellular injury from PM_2.5_ and O_3_ exposure compared to that in the general population. Co-exposure to high levels of PM_2.5_ and O_3_ had an antagonistic effect on the elevation of hepatic enzymes concentrations. Effective policies should be adopted to reduce air pollutant concentrations so as to minimize the liver damage in PWHA. These findings highlight the relationship between air pollutants and liver function in PWHA, providing a scientific basis for the implementation of measures to protect susceptible populations from the adverse effects of air pollution.

## Figures and Tables

**Table 1:** Characteristics of subjects included in the study at baseline (*n* = 137).

Characteristics	*n* (%)/Mean ± SD
Age (years)	47.64 ± 14.93
Sex	
Male	128 (93.43)
Female	9 (6.57)
BMI (kg/m^2^)	22.13 ± 2.88
Occupation	
Office worker	71 (51.82)
Manual worker	26 (18.98)
Other	40 (29.20)
Education	
Junior	39 (28.46)
Senior	32 (23.36)
College or above	66 (48.18)
Marital status	
Unmarried	55 (40.15)
Divorced	24 (17.52)
Married	58 (42.33)
Annual income	
<RMB 50,000	73 (53.28)
RMB 50,000–100,000	43 (31.39)
>RMB 100,000	21 (15.33)
Smoking history	
Current	33 (24.09)
Former	21 (15.33)
Never	83 (60.58)
Drinking status	
Current	28 (20.44)
Former	18 (13.14)
Never	91 (66.42)
Disease history	
No	89 (64.96)
Hypertension	23 (16.79)
Diabetes	8 (5.84)
Cardiovascular disease	7 (5.11)
Other diseases	10 (7.30)
ALT (U/L)	30.52 ± 24.41
AST(U/L)	69.54 ± 25.95
ALP (U/L)	96.08 ± 24.98
γ-GGT (U/L)	52.62 ± 39.91

Note: SD, standard deviation; BMI, body mass index; ALT, alanine aminotransferase; AST, aspartate aminotransferase; ALP, alkaline phosphatase; γ-GGT, gamma-glutamyl transferase.

**Table 2:** The 7-day average levels of pollutants and ambient temperature for 137 participants throughout the follow-up period.

Variables	Mean	SD	IQR	Min	Max
PM_2.5_ (µg/m^3^)	39.8	27.0	37.5	6.6	96.0
O_3_ (µg/m^3^)	100.7	24.1	33.7	53.1	136.6
SO_4_^2−^ (µg/m^3^)	5.5	3.6	5.4	0.8	15.7
NH_4_^+^ (µg/m^3^)	4.2	3.2	6.0	0.4	12.3
NO_3_^−^ (µg/m^3^)	6.7	5.2	9.5	0.5	18.3
OM (µg/m^3^)	11.9	8.9	10.4	1.1	31.7
BC (µg/m^3^)	2.1	1.4	1.8	0.2	5.2
Temperature (°C)	19.0	8.5	9.3	3.6	31.6

Note: PM_2.5_, particulate matter with an aerodynamic diameter of ≤2.5 µm; O_3_, ozone; SO_4_^2−^, sulfate; NO_3_^−^, nitrate; NH_4_^+^, ammonium; BC, black carbon; OM, organic matter; SD, standard deviation; IQR, interquartile range.

**Table 3:** Interaction of exposure to PM_2.5_ and O_3_ on levels of hepatic enzymes.

Category	% Changes in Hepatic Enzymes and 95%CI			
	ALT	AST	ALP	γ-GGT
PM_2.5_low-O_3_low	Reference	Reference	Reference	Reference
PM_2.5_low-O_3_high	3.43 (−11.36, 20.69)	19.58 (8.41, 31.90)	−2.22 (−6.73, 2.51)	0.19 (−9.43, 10.83)
PM_2.5_high-O_3_low	29.80 (−3.11, 73.89)	36.42 (13.59, 63.84)	−6.77 (−14.87, 2.09)	8.40 (−10.72, 31.62)
PM_2.5_high-O_3_high	12.06 (−6.41, 34.19)	31.79 (17.57, 47.74)	−3.24 (−8.44, 2.26)	6.66 (−5.23, 20.03)
Synergy index	0.363	0.568	0.360	0.775

Note: PM_2.5_, particulate matter with an aerodynamic diameter of ≤2.5 µm; O_3_, ozone; ALT, alanine aminotransferase; AST, aspartate aminotransferase; ALP, alkaline phosphatase; γ-GGT, gamma-glutamyl transferase.

## Data Availability

The datasets used or analyzed during the current study are available from the corresponding author on reasonable request.

## Supplementary Materials

The following supporting information can be downloaded at: https://www.mdpi.com/article/10.3390/toxics11090729/s1: Figure S1 The flow chart of participants’ recruitment and follow-up visits. Table S1 Associations of short-term exposure to PM_2.5_ with percent change in hepatic enzymes per 10 μg/m^3^ of exposure concentration in PWHA with no disease history. Table S2 Associations of short-term exposure to O_3_ with percent change in hepatic enzymes per 10 μg/m^3^ of exposure concentration in PWHA with no disease history. Table S3 Associations of short-term exposure to PM_2.5_ with percent change in hepatic enzymes per 10 μg/m^3^ of exposure concentration in PWHA without alcohol consumption. Table S4 Associations of short-term exposure to O_3_ with percent change in hepatic enzymes per 10 μg/m^3^ of exposure concentration in PWHA without alcohol consumption.
