# Supplementary material for: Short-Term Exposure to PM2.5 and O3 Impairs Liver Function in HIV/AIDS Patients: Evidence from a Repeated Measurements Study
Source: Toxics. 2023 Aug 25;11(9):729. doi: 10.3390/toxics11090729 (PMC10537338; doi:10.3390/toxics11090729)
Supplement: Supplementary file 1 [file toxics-11-00729-s001.zip › toxics-2542080-supplementary.pdf]

Short-Term Exposure to $PM_{2.5}$ and O_3 Impairs Liver Function in HIV/AIDS Patients: Evidence From a Repeated Measurements Study

Table of Contents

Figure S1 The flow chart of participant recruitment and follow-up visits.

Table S1 Associations of short-term exposure to $PM_{2.5}$ with percent change in hepatic enzymes per 10μg/m^3 of exposure concentration in PWHA with no disease history.

Table S2 Associations of short-term exposure to O_3 with percent change in hepatic enzymes per 10μg/m^3 of exposure concentration in PWHA with no disease history.

Table S3 Associations of short-term exposure to $PM_{2.5}$ with percent change in hepatic enzymes per 10μg/m^3 of exposure concentration in PWHA without alcohol comsumption.

Table S4 Associations of short-term exposure to O_3 with percent change in hepatic enzymes per 10μg/m^3 of exposure concentration in PWHA without alcohol comsumption.

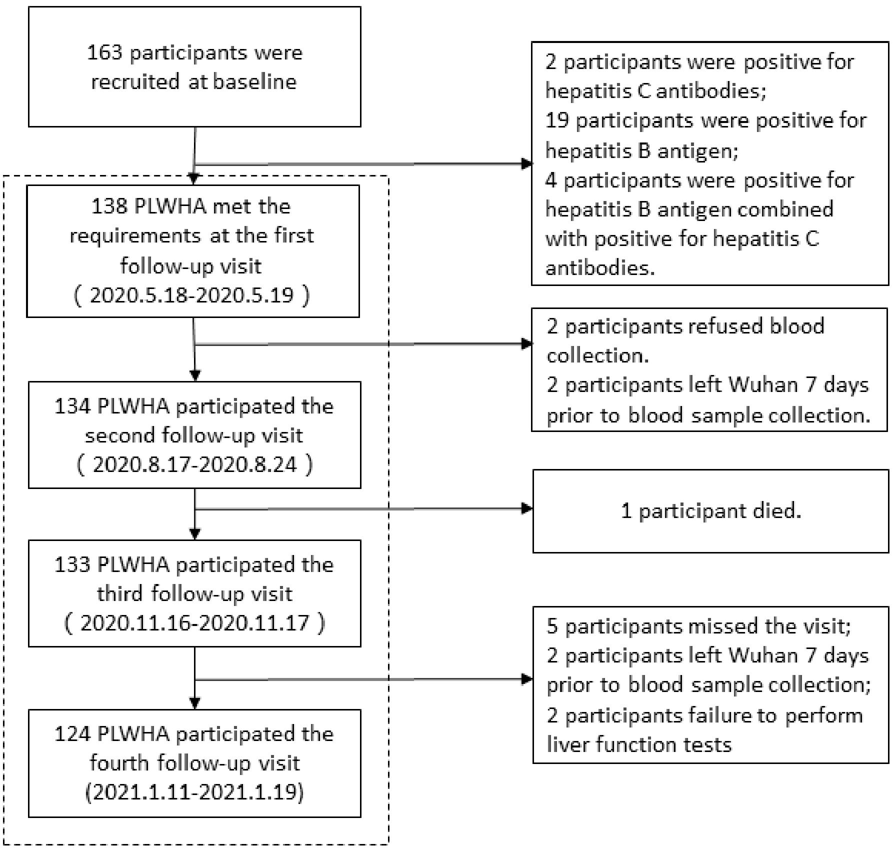

Figure S1. The flow chart of participant recruitment and follow-up visits.

Table S1. Associations of short-term exposure to $PM_{2.5}$ with percent change in hepatic enzymes per 10μg/m^3 of exposure concentration in PWHA with no disease history.

Exposure	% Changes in ALT, U/L	% Changes in AST, U/L	% Changes in ALP U/L	% Changes in γ-GGT, U/L
	β (95%CI)	β (95%CI)	β (95%CI)	β (95%CI)
lag0	2.53 (-1.76 to 7.03)	8.93 (5.92 to 11.83)	0.03 (-1.22 to 1.32)	0.64 (-2.25 to 3.59)
lag1	0.51 (-3.62 to 4.81)	4.26 (1.58 to 6.82)	-0.49 (-1.70 to 0.73)	0.09 (-2.71 to 2.94)
lag2	1.45 (-1.38 to 4.33)	7.26 (4.40 to 9.07)	-0.63 (-1.43 to 0.18)	0.15 (-1.75 to 2.02)
lag3	1.43 (-1.36 to 4.18)	5.12 (3.22 to 6.85)	-0.19 (-0.98 to 0.61)	-0.14 (-1.98 to 1.71)
lag4	0.77 (-1.92 to 3.54)	4.35 (2.73 to 5.99)	-0.37 (-1.18 to 0.45)	-0.10 (-2.00 to1.77)
lag5	1.75 (-0.97 to 4.50)	0.69 (-1.06 to 2.32)	0.65 (-0.16 to1.04)	0.98 (-0.89 to 2.85)
lag6	2.56 (-2.89 to 8.59)	6.37 (2.51 to 10.37)	-0.14 (-0.15 to 1.48)	2.43 (-1.33 to 6.32)
lag01	1.34 (-3.46 to 6.37)	6.64 (3.43 to 9.73)	0.14 (-1.27 to 1.58)	0.74 (-2.53 to 4.10)
lag02	1.49 (-3.24 to 6.42)	8.70 (5.59 to 11.68)	-0.23 (-1.60 to 1.17)	0.79 (-2.43 to 4.05)
lag03	1.58 (-3.16 to 6.45)	8.56 (5.44 to 11.45)	-0.15 (-1.53 to 1.25)	0.78 (-2.45 to 4.04)
lag04	0.94 (-3.43 to 5.44)	7.62 (4.77 to 10.23	-0.36 (-1.65 to 0.94)	0.33 (-2.69 to 3.36)
lag05	2.20 (-3.28 to 7.87)	9.08 (5.43 to 12.38)	0.23 (-1.39 to 1.88)	1.29 (-2.53 to 5.12)
lag06	2.66 (-3.38 to 8.97)	10.08 (6.03 to 13.80)	0.30 (-1.49 to 2.13)	1.97 (-2.26 to 6.23)

Note: All analyses were adjusted for sex, age, BMI, education, occupation, marital status, annual income, smoking history, alcohol consumption, and temperature. HIV, human immunodeficiency virus; $PM_{2.5}$, particulate matter with an aerodynamic diameter of≤2.5 um; PWHA, people living with HIV/AIDS; CI, confidence interval; ALT, alanine aminotransferase; AST, aspartate aminotransferase; ALP, alkaline phosphatase; γ-GGT, gamma-glutamyl transferase.

Table S2. Associations of short-term exposure to O_3 with percent change in hepatic enzymes per 10μg/m^3 of exposure concentration in PWHA with no disease history.

Exposure	% Changes in ALT, U/L	% Changes in AST, U/L	% Changes in ALP U/L	% Changes in γ-GGT, U/L
	β (95%CI)	β (95%CI)	β (95%CI)	β (95%CI)
	O_3 (μg/m^3)			
lag0	7.67 (0.26 to 15.94)	20.74 (15.57 to 26.44)	-0.46(-2.54 to -1.68)	2.70 (-2.20 to 7.80)
lag1	3.37 (-3.60 to 10.94)	1.99 (-2.22 to 6.54)	1.66 (-0.39 to 3.76)	1.45 (-3.18 to 6.43)

lag2	1.04 (-4.24 to 6.89	-8.18 (-11.07 to -4.68)	0.94 (-0.69 to 2.63)	-0.69 (-4.31to 3.19)
lag3	1.76 (-3.44 to 7.99)	3.61 (0.04 to 7.31)	0.78 (-0.73 to 2.34)	2.55 (-0.98 to 6.26)
lag4	2.48 (-0.28 to 5.25)	8.92 (7.38 to 10.47)	-0.73 (-1.51 to 0.04)	0.42 (-1.41 to 2.21)
lag5	2.89 (-1.83 to 7.74)	6.46 (3.37 to 9.50)	0.10 (-1.25 to 1.46)	1.07 (-2.07 to 4.24)
lag6	3.46 (-0.58 to 7.61)	12.34 (9.63 to 15.01)	-0.94 (-2.06 to 0.19)	0.26 (-2.39 to 2.92)
lag01	6.84 (-1.62 to 16.26)	16.39 (10.78 to 22.52)	1.11 (-1.32 to 3.61)	3.02 (-2.61 to 9.00)
lag02	5.88 (-2.89 to 15.86)	4.33 (-0.93 to 10.37)	1.86 (-0.76 to 4.58)	1.55 (-4.36 to 7.97)
lag03	6.40 (-2.41 to 16.35)	6.75 (1.31 to 12.75)	1.88 (-0.71 to 4.58)	2.99 (-2.96 to 9.45)
lag04	6.13 (-1.58 to 14.58)	15.42 (10.49 to 20.68)	0.59 (-1.61 to 2.85)	2.54 (-2.59 to 7.95)
lag05	6.54 (-1.27 to 15.07)	17.65 (12.60 to 23.02)	0.49 (-1.71 to 2.76)	2.59 (-2.58 to 7.99)
lag06	6.21 (-1.14 to 14.16)	18.41 (13.64 to 23.41)	0.07 (-1.99 to 2.18)	2.06 (-2.78 to 7.08)

Note: All analyses were adjusted for sex, age, BMI, education, occupation, marital status, annual income, smoking history, alcohol consumption, and temperature. HIV, human immunodeficiency virus; O_3, ozone; PWHA, people living with HIV/AIDS; CI, confidence interval; ALT, alanine aminotransferase; AST, aspartate aminotransferase; ALP, alkaline phosphatase; γ-GGT, gamma-glutamyl transferase.

Table S3. Associations of short-term exposure to $PM_{2.5}$ with percent change in hepatic enzymes per 10μg/m^3 of exposure concentration in PWHA without alcohol comsumption.

Exposure	% Changes in ALT, U/L	% Changes in AST, U/L	% Changes in ALP U/L	% Changes in γ-GGT, U/L
	β (95%CI)	β (95%CI)	β (95%CI)	β (95%CI)
	PM_{25} (μg/m^3)			
lag0	0.25 (-3.21 to 3.88)	5.76 (3.44 to 8.13)	0.44 (-0.70 to 1.65)	-0.10 (-2.31 to 2.16)
lag1	-1.51 (-4.93 to 2.10)	3.29 (1.10 to 5.59)	-0.95 (-2.07 to 0.23)	-1.84 (-4.02 to 0.37)
lag2	0.13 (-2.45 to 2.81)	6.53 (4.86 to 8.29)	-0.93 (-1.76 to -0.06)	-0.27 (-1.92 to 1.39)
lag3	0.06 (-2.67 to 2.90)	2.89 (1.12 to 4.76)	-0.82 (-1.71 to 0.10)	-0.73 (-2.46 to 1.05)
lag4	0.52 (-1.78 to 2.99)	2.82 (1.43 to 4.32)	-0.25 (-1.03 to 0.55)	0.06 (-1.46 to 1.61)
lag5	1.40 (-0.72 to 3.55)	1.20 (-0.22 to 2.57)	0.37 (-0.32 to 1.06)	0.50 (-0.82 to 1.82)
lag6	2.88 (-2.35 to 8.65)	6.34 (2.74 to 10.23)	0.65 (-1.03 to 2.48)	-0.59 (-3.85 to 2.81)
lag01	-1.22 (-5.11 to 2.90)	4.49 (1.93 to 7.14)	0.05 (-1.25 to 1.43)	-0.96 (-3.48 to 1.62)
lag02	-0.88 (-4.85 to 3.32)	6.34 (3.76 to 9.01)	-0.42 (-1.74 to 0.97)	-0.67 (-3.25 to 1.96)
lag03	-0.77 (-5.02 to 3.76)	6.32 (3.58 to 9.17)	-0.79 (-2.20 to 0.71)	-0.87 (-3.65 to 1.97)

lag04	-0.93 (-4.91 to 3.78)	5.58 (3.05 to 8.24)	-0.89 (-2.23 to 0.53)	-0.83 (-3.46 to 1.87)
lag05	0.19 (-4.63 to 5.39)	6.82 (3.71 to 10.01)	-0.34 (-1.95 to 1.38)	-0.18 (-3.36 to 3.09)
lag06	0.65 (-4.47 to 6.20)	7.61 (4.26 to 11.05)	-0.13 (-1.84 to 1.70)	-0.14 (-3.50 to 3.33)

Note: All analyses were adjusted for sex, age, BMI, education, occupation, marital status, annual income, smoking history, disease history, and temperature. HIV, human immunodeficiency virus; $PM_{2.5}$, particulate matter with an aerodynamic diameter of≤2.5 um; PWHA, people living with HIV/AIDS; CI, confidence interval; ALT, alanine aminotransferase; AST, aspartate aminotransferase; ALP, alkaline phosphatase; γ-GGT, gamma-glutamyl transferase.

Table S4. Associations of short-term exposure to O_3 with percent change in hepatic enzymes per 10μg/m^3 of exposure concentration in PWHA without alcohol consumption.

Exposure	% Changes in ALT, U/L	% Changes in AST, U/L	% Changes in ALP U/L	% Changes in γ-GGT, U/L
	β (95%CI)	β (95%CI)	β (95%CI)	β (95%CI)
	O_3 (μg/m^3)			
lag0	8.12 (1.71 to 15.11)	19.59 (15.38 to 24.20)	0.41 (-1.16 to 2.46)	2.96 (-0.93 to 7.07)
lag1	5.41 (-1.10 to 12.36)	3.06 (-0.95 to 7.30)	1.69 (-0.02 to 3.80)	3.44 (-0.55 to 7.63)
lag2	2.93 (-1.83 to 8.15)	-9.53 (-12.4 to -6.67)	0.74 (--0.00 to 2.38)	0.83 (-2.15 to 4.00)
lag3	2.76 (-2.17 to 7.76)	8.90 (5.595 to 12.20)	0.17 (-0.01 to 1.77)	1.00 (-2.03 to 4.09)
lag4	2.07 (-0.58 to 4.77)	8.65 (7.164 to 10.14)	-0.94 (-0.05 to -0.09)	0.32 (-1.33 to 1.99)
lag5	2.69 (-1.12 to 6.81)	5.72 (3.17 to 8.37)	-0.64 (-1.10 to 0.62)	1.48 (-0.92 to 3.96)
lag6	4.51 (0.51 to 8.78)	12.48 (9.809 to 15.17)	-1.20 (-2.24 to 0.07)	0.80 (-1.64 to 3.35)
lag01	8.43 (0.71 to 16.89)	17.62 (12.62 to 23.16)	1.78 (-1.14 to 4.26)	4.63 (-0.11 to 9.65)
lag02	8.48 (0.25 to 17.77)	4.41 (-0.59 to 10.03)	2.28 (-0.09 to 5.01)	4.28 (-0.78 to 9.75)
lag03	8.82 (0.34 to 18.17)	10.42 (5.126 to 16.24)	1.94 (-0.07 to 4.71)	4.10 (-1.07 to 9.63)
lag04	6.62 (-0.67 to 14.50)	18.45 (13.83 to 23.42)	0.46 (-0.05 to 2.83)	3.04 (-1.45 to 7.78)
lag05	6.38 (-0.79 to 14.20)	18.70 (14.01 to 23.70)	0.10 (-1.13 to 2.44)	3.26 (-1.19 to 7.95)
lag06	6.28 (-0.56 to 13.74)	18.67 (14.29 to 23.41)	-0.25 (-1.16 to 1.97)	2.81 (-1.42 to 7.27)

Note: All analyses were adjusted for sex, age, BMI, education, occupation, marital status, annual income, smoking history, disease history, and temperature. HIV, human immunodeficiency virus; O_3, ozone; PWHA, people living with HIV/AIDS; CI, confidence interval; ALT, alanine aminotransferase; AST, aspartate aminotransferase; ALP, alkaline phosphatase; γ-GGT, gamma-glutamyl transferase.
